# Effects of cognitive rehabilitation and exercise on brain structure in progressive multiple sclerosis: results from the CogEx trial

**DOI:** 10.1007/s00415-025-13382-9

**Published:** 2025-09-23

**Authors:** Francesco Romanò, Maria A. Rocca, Elisabetta Pagani, Maria Pia Amato, Giampaolo Brichetto, Jeremy Chataway, Nancy D. Chiaravalloti, Gary Cutter, Ulrik Dalgas, John DeLuca, Rachel Farrell, Peter Feys, Jennifer Freeman, Matilde Inglese, Emilio Cipriano, Cecilia Meza, Robert W. Motl, Amber Salter, Brian M. Sandroff, Anthony Feinstein, Massimo Filippi, CogEx Research Team

**Affiliations:** 1 Neuroimaging Research Unit, Division of Neuroscience, IRCCS San Raffaele Scientific Institute, Via Olgettina, 60, 20132 Milan, Italy; 2 Neurology Unit, IRCCS San Raffaele Scientific Institute, Milan, Italy; 3 Vita-Salute San Raffaele University, Milan, Italy; 4 Department NEUROFARBA, Section Neurosciences, University of Florence, Florence, Italy; 5 IRCCS Fondazione Don Carlo Gnocchi, Florence, Italy; 6 Scientific Research Area, Italian Multiple Sclerosis Foundation (FISM), Genoa, Italy; 7 AISM Rehabilitation Service, Italian Multiple Sclerosis Society, Genoa, Italy; 8 Faculty of Brain Sciences, Queen Square Multiple Sclerosis Centre, Department of Neuroinflammation, UCL Queen Square Institute of Neurology, University College London, London, WC1B 5EH UK; 9 National Institute for Health Research, University College London Hospitals, Biomedical Research Centre, London, W1T 7DN UK; 10 Kessler Foundation, West Orange, NJ USA; 11 Department of Physical Medicine and Rehabilitation, Rutgers NJ Medical School, Rutgers University Newark, Newark, NJ USA; 12 Department of Biostatistics, University of Alabama at Birmingham, Birmingham, AL USA; 13 Exercise Biology, Department of Public Health, Aarhus University, Aarhus, Denmark; 14 Faculty of Rehabilitation Sciences, REVAL, Hasselt University, Diepenbeek, Belgium; 15 UMSC Hasselt, Pelt, Belgium; 16 Faculty of Health, School of Health Professions, University of Plymouth, Devon, UK; 17 Department of Neuroscience, Rehabilitation, Ophthalmology, Genetics, Maternal and Child Health, and Center of Excellence for Biomedical Research, University of Genoa, Genoa, Italy; 18 IRCCS Ospedale Policlinico San Martino, Genoa, Italy; 19 Department of Psychiatry, University of Toronto and Sunnybrook Health Sciences Centre, Toronto, Canada; 20 Department of Kinesiology and Nutrition, University of Illinois Chicago, Chicago, IL USA; 21 Department of Neurology, Section on Statistical Planning and Analysis, UT Southwestern Medical Center, Dallas, TX USA; 22 Neurorehabilitation Unit, IRCCS San Raffaele Scientific Institute, Milan, Italy; 23 Neurophysiology Service, IRCCS San Raffaele Scientific Institute, Milan, Italy

**Keywords:** Multiple Sclerosis, Magnetic Resonance Imaging, Exercise Therapy, Cognitive Rehabilitation, Voxelwise, Neuroplasticity

## Abstract

**Background:**

We previously showed increased cortical grey matter (GM) volume in CogEx trial participants who performed cognitive rehabilitation (CR). Here, we explore combined CR and aerobic exercise (EX) effects on regional changes in brain volumes and white matter (WM) integrity.

**Methods:**

Seventy-three patients were randomized into four groups receiving a combination of CR and EX or their sham versions: CR + EX, CR + EX-sham, EX + CR-sham, and CR-sham + EX-sham. A diagnosis of progressive multiple sclerosis (PMS) and impaired information processing speed were required for inclusion. Participants attended a 12-week intervention twice/week. Assessments were performed at baseline, week-12 (W12), and nine months post-baseline (M9). Structural MRI scans were acquired with a standardized protocol, and voxelwise variations of brain volumes and WM fractional anisotropy (FA) were analyzed.

**Results:**

Baseline regional brain volumes and WM FA were comparable between groups. Voxelwise analyses at W12 and M9 revealed generalized volume reductions in all groups. We found different patterns of volumetric changes in the left inferior temporal gyrus between CR + EX and CR-sham + EX-sham, and in the right cerebellum crus II between EX + CR-sham and CR + EX-sham. WM FA values remained stable throughout the trial and no longitudinal between-group differences were found.

**Conclusions:**

Our analysis showed a decrease in brain volumes and limited effects of the combined CR + EX intervention, indicating that the previously found cortical GM increase was not superimposable at voxel level. Methodological and sampling differences between the studies could explain these discrepancies. In few cognitively relevant areas, the combined CR interventions might have affected patterns of volume changes, while EX modified cerebellar motor regions.

**Clinical trial registration:**

The main trial was registered on ClinicalTrials.gov (NCT03679468; registration date: 20 Sep 2018).

**Supplementary Information:**

The online version contains supplementary material available at 10.1007/s00415-025-13382-9.

## Introduction

Multiple sclerosis (MS) is an inflammatory and neurodegenerative disease affecting the central nervous system with heterogeneous clinical manifestations. Cognitive impairment is highly prevalent in people with MS, and the proportion of affected patients is greater in progressive phenotypes [1]. Compared with cognitively preserved patients, those with any degree of cognitive impairment tend to be older, have MS for more years, and present more widespread structural damage, in terms both of focal white matter (WM) lesions and atrophy [2]. These alterations were found to be more pronounced in patients with more severe deficits or with an involvement of multiple cognitive domains [2, 3]. In recent years, the possibility of improving cognitive dysfunction with cognitive rehabilitation and physical exercise and exploring the response of the central nervous system to such stimuli has gained interest. The combination of these approaches has been studied mainly in relapsing–remitting MS, while effects in patients with progressive MS (PMS) and greater disease severity have not been investigated systematically [4].

The CogEx study [5] assessed whether there is a synergistic effect on cognitive functioning when both cognitive rehabilitation (CR) and aerobic exercise (EX) are administered to patients with PMS and impaired processing speed. This was expected to contribute to a better understanding of the application of both interventions in a subgroup where impaired brain plasticity and reserve could represent limiting factors [6, 7].

The analyses conducted up to now after the end of the trial [5, 8] showed that performing CR and EX in combination did not improve processing speed any better than single or sham interventions. However, a clinically meaningful cognitive improvement, defined as an increase of more than 4 points on the Symbol Digit Modalities Test (SDMT) following treatment, was observed in a substantial proportion of patients, regardless of the intervention type, with 60% of all participants demonstrating this improvement [5]. On the other hand, the analysis of structural and functional magnetic resonance imaging (MRI) data highlighted differential effects between treatments, with CR resulting in increased global cortical grey matter (GM) volume and increased activity of several areas during a cognitive task [8]. We also showed that a significant increase of cortical GM volume was observed in frontal, parietal, and temporal lobes, indicating that intervention effects might be specific to brain regions that were more involved during training [8].

In this context, voxelwise MRI analyses can be employed to better characterize localized structural modifications in brain GM and WM. These methodologies have been used to assess neurodegeneration in MS [9–11], which is more pronounced and widespread in patients with PMS [12], and to study neuroplastic adaptations following rehabilitation [13, 14]. Potential structural changes following rehabilitation have been hypothesized to occur as a result of angiogenesis, dendrite pruning, remyelination, decrease of inflammation level and consequent change of the microstructure [15, 16]. Whether these effects are localized and whether they are persistent once rehabilitation is finished is uncertain. It is also unknown how these mechanisms interact with the progressive neurodegenerative damage that characterizes people with PMS.

To address these points, we describe the explorative analysis of GM and WM modifications at voxel level within the CogEx MRI substudy both in volume and in microstructure. Our objective was to evaluate the effect of the combined CR and EX intervention, as well as the single treatment components, on these outcomes, and to study whether regional structural neuroplasticity represents a possible substrate of the changes in cognitive performance observed in the whole sample. We hypothesized that structural adaptations would be evident in cortical regions/WM tracts connected to the stimuli provided during treatment, and that, given the aim for which the CogEx trial was designed, the most prominent associations with cognitive Changes after treatment would be found in the combined CR and EX group. Considering previous reports showing Changes in both WM integrity and brain volumes after 12 weeks of CR and EX (alone or combined) in a healthy aging sample, we assumed this timeframe to be appropriate also for detecting structural neuroplasticity in our study [17, 18].

## Materials and methods

### Study design and participants

The CogEx trial was a randomized sham-controlled trial. After screening, the baseline assessment was performed, and patients were randomized to one of four treatment arms (1:1:1:1 ratio): CR + EX; CR + EX-sham; EX + CR-sham and CR-sham + EX-sham. Then, they underwent 12 weeks of intervention, twice per week, and performed follow-up assessments at the end of the intervention (W12) and nine months from the baseline assessment (M9). Of the 11 centres involved in the CogEx trial, four participated in the MRI substudy: (a) IRCCS San Raffaele Hospital (Milan, Italy); (b) University of Genoa (Genoa, Italy); (c) University of Alabama at Birmingham (Birmingham, Alabama, USA) and d) Kessler Foundation (East Hanover, New Jersey, USA).

Patients were enrolled between 14th Dec 2018 and 2nd April 2022. Complete inclusion and exclusion criteria of the CogEx trial are reported elsewhere [5, 19]. Importantly, patients were required to have a confirmed diagnosis of PMS, age between 25 and 65 years, an Expanded Disability Status Scale (EDSS) score lower than 7.0, and impaired information processing speed according to their performance in the SDMT (below the 10th percentile of published normative data in each country). Participants were excluded if they performed habitual aerobic exercise, had undergone treatment with steroids in the 3 months prior and had a history of substance abuse or severe mental illness. Additionally, as specific criteria for the analyses performed in the current study, participants were required to have complete neuropsychological and structural MRI assessments at all three timepoints, with sufficient image quality in either T1-weighted or diffusion-weighted MRI scans.

### Interventions

Full details regarding the interventions, including information on duration, content, modality, and progression of each treatment component have been reported extensively in the appendix of the main publication [5]. Briefly, CR was provided with the computerized RehaCom program using modules of divided and sustained attention, concentration, and vigilance. CR-sham consisted of Internet training with durations of personnel contact and computer usage matched with the CR group. EX consisted of aerobic exercise performed on a recumbent arm-leg step ergometer (NuStep T5XR, Ann Arbor, MI, USA), alternating each session between moderate-intensity continuous training and high-intensity interval training. EX-sham was focused on balance training and stretching, designed specifically to avoid any cardiovascular effort and any cognitive-motor dual tasking. While the duration of CR and CR-sham sessions was fixed at around 40 min, the duration of EX and EX-sham session increased progressively throughout the trial from 20 to 60 min. After the end of the 12 weeks of intervention no additional treatment, apart from the usual care, was provided.

### MRI outcomes

#### Acquisition protocol

Using 3.0 Tesla scanners (IRCCS San Raffaele: Philips Ingenia CX; University of Genoa and University of Alabama: Siemens Prisma; Kessler Foundation: Siemens Skyra) and standardized guidelines for participants’ positioning, the following brain MRI sequences were acquired: a) variable flip angle 3D T2-weighted fluid-attenuated inversion recovery (FLAIR) turbo spin echo (Philips scanner: repetition time [TR] = 4800 ms; echo time [TE] = 270 ms; inversion time [TI] = 1650 ms; matrix size = 256 × 256; field of view [FOV] = 256 × 256 mm^2^; echo train length [ETL] = 167; 192 contiguous sagittal slices, 1 mm thick; Siemens scanners: TR = 5000 ms; TE = 395 ms; TI = 1800 ms; matrix size = 256 × 256; FOV = 256 × 256 mm^2^; ETL = 284; 192 contiguous sagittal slices, 1.05 mm thick), b) sagittal 3D T1-weighted sequence: (Philips scanner: TR = 7 ms; TE = 3.2 ms; TI = 1000 ms; flip angle = 8°; matrix size = 256 × 256; FOV = 256 × 256 mm^2^; 204 contiguous sagittal slices, 1 mm thick; Siemens scanners: TR = 2300 ms; TE = 2.98 ms; TI = 900 ms; flip angle = 9°; matrix size = 256 × 256; FOV = 256 × 256 mm^2^; 204 contiguous sagittal slices, 1 mm thick); and c) axial pulsed-gradient spin echo single shot diffusion-weighted echo planar imaging (EPI) (all scanners: 3 shells at b-value = 700/1000/2855 s/mm^2^ along 6/30/60 non-collinear directions and 10 b = 0 volumes were acquired, FOV = 240 × 233 mm, pixel size = 2.14 × 2.69 mm, 56 slices, 2.3 mm-thick, matrix = 112 × 85, TR = about 6000 ms, TE = about 80 ms and three additional b = 0 volumes with reversed polarity of gradients for distortion correction).

#### Conventional MRI analysis

T2-hyperintense lesions were identified on baseline 3D FLAIR scans using an automated segmentation approach and their volume (LV) was obtained. Normalized brain, GM and WM volumes (NBV, NGMV and NWMV) were extracted from lesion-filled 3D T1-weighted scans at baseline. Detailed processing steps are described elsewhere [8].

#### Voxel-based and tensor-based morphometry

Tensor-based morphometry (TBM), as implemented in SPM12, was used to map changes of regional brain volumes over time. Longitudinal registration was used to align each patients’ lesion-filled scans to a mid-point average template [20], which was then used for iterative groupwise alignment using the Diffeomorphic Anatomical Registration Through Exponentiated Lie algebra (DARTEL) method [21]. Finally, an affine transformation that maps from the population average (DARTEL Template space) to Montreal Neurological Institute (MNI) space was calculated, the rate of longitudinal volume changes (difference of jacobians of the deformation) were spatially normalized to MNI and smoothed with an 8 mm Gaussian kernel.

The steps described for groupwise alignment were repeated for baseline 3D T1-weighted images to run a voxel-based morphometry (VBM) analysis. The only difference in the procedure described above is that normalization to MNI space was applied to brain maps.

#### Tract-based spatial statistics

Preprocessing of diffusion-weighted imaging data included correction for off-resonance and eddy current induced distortions, using the Eddy tool within the FSL library [22].

The diffusion tensor (DT) was estimated in each voxel using the shell at b ≤ 1000 s/mm^2^ by linear regression [23] using the FMRIB’s Diffusion Toolbox.

A longitudinal pipeline free of interpolation asymmetries was applied [24] using the spatial normalization methods [25] supported by the DTI-TK toolkit: first an unbiased within-subject template was generated from all the DT volumes of each patient, which was then used to produce a study specific template [26]. Fractional anisotropy (FA) maps from the population specific DTI template and from the transformed individual DTI were derived. Finally, a tract-based spatial statistics (TBSS) analysis [27] was used to perform a voxelwise analysis of whole-brain WM FA. In detail, the population FA template was thinned to create a WM tract “skeleton”, which was thresholded at FA > 0.2 to include only WM voxels. Individual-subject FA values were projected onto this group skeleton by searching perpendicular from the skeleton for maximum FA value.

For statistical analysis of differences between changes at W12 vs baseline and changes at M9 vs W12, skeletonized FA values of the earlier time-point were subtracted from those of the subsequent one (“W12—baseline” and “M9—W12”).

### Clinical and neuropsychological outcomes

Clinical and neuropsychological assessments were performed at baseline, W12, and M9 by assessors blinded to treatment allocation.

Cognitive performances were assessed with the Brief International Cognitive Assessment of Multiple Sclerosis (BICAMS), a reliable and sensitive measure of cognition in people with MS [28], which includes the SDMT for processing speed evaluation, the Brief Visuospatial Memory Test Revised (BVMT-R) for visual memory evaluation and the California Verbal Learning Test-II (CVLT-II) for verbal memory evaluation. Age-, sex-, and education-adjusted z-scores for cognitive tests were computed according to country-specific normative values.

Expanded Disability Status Scale (EDSS) scores were provided by each participant’s treating neurologist at baseline only. Also, evaluations of walking capacity (6-min walking test), physical activity and cardio-respiratory fitness were performed at all time points.

Complete information regarding the methodology of each evaluation is reported in the protocol paper [19].

### Statistical analysis

Statistical analysis was performed using SPSS (IBM, version 26.0) for demographic and clinical data, SPM12 for voxelwise volumetric data, and FSL randomise for voxelwise diffusivity data.

Descriptive statistics were reported as means (standard deviation [SD]) or median (interquartile range [IQR]) for continuous variables, while categorical variables were reported as frequencies. Between-group comparisons were performed using Chi-square, ANOVA (with Bonferroni-corrected post-hoc tests), and Kruskal–Wallis tests as appropriate.

Volumetric and diffusivity Changes were assessed within and between groups using one-way ANOVAs with a 4-levels factor adjusting for acquisition center. All comparisons were corrected for age and sex. For TBSS analysis, a permutation-based inference for non-parametric statistical thresholding was used, with number of permutations = 5000 and threshold-free cluster enhancement (TFCE) applied. For comparisons between changes at W12 vs baseline and changes at M9 vs W12, correction for follow-up length was applied. Additionally, in the TBM model a 2-level within-group factor for period was added, whereas for TBSS the difference between FA changes at W12 vs baseline and changes at M9 vs W12 was computed and analyzed. All comparisons were examined at the FWE-corrected threshold (*p* < 0.05) and at the uncorrected threshold (*p* < 0.001), with a cluster extent threshold of 10 voxels. Sensitivity analyses grouping CR vs CR-sham and EX vs EX-sham were also performed for all between-group comparisons.

Age- and sex-corrected multiple linear regression models were used to assess correlations between longitudinal volumetric/FA changes and changes in cognitive outcomes.

## Results

### Sample characteristics

A total of 73 patients with valid MRI and neuropsychological data at all time points were included in the current analysis. Due to the presence of movement artifacts some scans were not usable. Thus, 72 patients were included in the TBM analysis and 68 in the TBSS analysis.

Demographic, clinical, neuropsychological and conventional MRI baseline characteristics are reported in Table 1. There were significant differences between groups in CVLT z-score, which was higher in CR-sham + EX-sham than CR + EX-sham (*p* = 0.05), and in NBV, which was lower in CR + EX-sham than CR-sham + EX-sham (*p* = 0.002).

**Table 1:** Main demographic, clinical, neuropsychological and conventional MRI characteristics at baseline of multiple sclerosis (MS) patients included in this study, divided according to treatment allocation

	CR + EX	CR + EX-sham	EX + CR-sham	CR-sham + EX-sham	*P*
*N*	18	20	18	17	
Participants from Centers: San Raffaele/Genoa/Alabama/Kessler [*N*]	8/8/2/0	9/6/3/2	8/8/1/1	6/7/4/0	0.70^+^
Mean age [years] (SD)	50.4 (8.8)	52.7 (6.5)	52.5 (6.0)	52.2 (7.0)	0.77*
Sex (M/F)	9/9	7/13	6/12	4/13	0.43^+^
Median EDSS score (IQR)	5.25 (4.5–6.0)	5.25 (4.25–6.25)	5.75 (4.0–6.5)	6.0 (4.5–6.5)	0.71^++^
Mean disease duration [years] (SD)	12.7 (11.0)	17.0 (9.0)	15.4 (11.5)	19.8 (10.0)	0.23*
Type of MS (Primary/Secondary progressive)	7/11	4/16	4/14	2/15	0.28^+^
Mean 6MWT total distance [m] (SD)	232.1 (142.0)	256.6 (109.7)	224.8 (116.2)	282.2 (145.6)	0.77*
Mean VO_2_peak [ml/min/kg] (SD)	15.1 (5.4)	16.7 (6.6)	16.0 (4.6)	14.0 (6.5)	0.55*
Mean WR_peak_ [W] (SD)	73.3 (26.6)	76.0 (31.3)	74.1 (26.5)	75.0 (36.5)	0.99*
Mean average % in MVPA (SD)	1.7 (1.9)	1.4 (2.0)	2.2 (3.5)	1.4 (1.5)	0.74*
Mean education [total years of schooling] (SD)	12.2 (3.7)	13.9 (3.5)	14.1 (2.9)	14.6 (3.6)	0.17*
SDMT – mean number of correct responses (SD)	29.8 (7.4)	32.5 (6.2)	30.5 (5.9)	34.0 (9.2)	0.31*
Mean SDMT z-score (SD)	– 1.94 (0.5)	– 1.97 (0.7)	– 2.02 (0.6)	– 1.83 (0.4)	0.78*
Mean CVLT-II z-score (SD)	– 1.18 (0.9)	– 1.41 (1.0)	– 1.23 (1.0)	– 0.50 (1.1)	0.04*
Mean BVMT-R z-score (SD)	– 0.38 (0.9)	– 0.52 (1.4)	– 0.35 (1.1)	– 0.26 (0.7)	0.90*
Mean T2 LV [ml] (SD)	9.4 (8.9)	12.5 (10.9)	17.3 (11.5)	8.2 (8.9)	0.11*
Mean NBV [ml] (SD)	1485 (63)	1439 (52)	1471 (62)	1512 (60)	0.004*
Mean NGMV [ml] (SD)	815 (45)	792 (53)	803 (48)	833 (30)	0.05*
Mean NWMV [ml] (SD)	670 (34)	647 (25)	668 (43)	679 (44)	0.06*

Significant differences (*p* < 0.05) are highlighted in bold*ANOVA model; ^+^Chi-square test, ^++^Kruskall-Wallis test

### Clinical and neuropsychological outcomes

Full details regarding the longitudinal analysis of clinical and neuropsychological outcomes are reported in the main publication [5].

Briefly, there were no differences between groups regarding cognitive functions after treatment, although 171 (60%) of the 284 participants analyzed showed an improvement of at least 4 points on the SDMT at W12. Regarding aerobic fitness, there were significant improvements at W12 in EX versus EX-sham groups, which were not maintained at M9. No differences were observed in walking capacity or physical activity measures.

### Regional Volumetric analysis

At baseline, there were no regional volumetric differences between the four treatment arms (FWE-corrected threshold).

#### Longitudinal differences – W12 vs baseline

Within-groups volumetric changes from baseline to W12 showed a few clusters of increased volume and several clusters of decreased volume in occipital, temporal, frontal, parietal and cerebellar areas in all groups (uncorrected threshold).

Longitudinal volumetric changes from baseline to W12 were not significantly different between the four treatment arms (FWE-corrected threshold).

At the uncorrected threshold, there were significant effects of treatment on right lingual gyrus volume (increased in CR-sham + EX-sham compared with CR + EX-sham and CR + EX), left cerebellum lobule IX volume (decreased in CR-sham + EX-sham and CR + EX compared with EX + CR-sham), and right cerebellum lobule VIII volume (decreased in CR + EX compared with EX + CR-sham and CR-sham + EX-sham).

Detailed findings from this analysis are reported in Tables 2 and 3.

**Table 2:** Within-group longitudinal volumetric Changes at week 12 vs baseline

Comparison	kE	pFWE	T	MNIx	MNIy	MNIz	BA	Area
CR-sham + EX-sham increase	72	0.282	4.49	8	– 56	2	18	R Lingual Gyrus
	10	0.905	3.79	66	– 21	21	22	R SMG
	11	0.94	3.71	66	– 16	20	48	R SMG
	18	0.947	3.7	45	– 33	62	3	R Postcentral Gyrus
	14	0.982	3.57	– 18	– 22	– 24	–	L PHG
	23	0.998	3.39	4	– 9	63	6	R SMA
CR-sham + EX-sham decrease	256	0.331	4.43	– 9	– 42	– 44	–	L Cerebellum Lobule IX
	99	0.839	3.88	– 15	– 68	– 56	–	L Cerebellum Lobule VIII
	44	0.933	3.73	39	– 52	– 46	–	R Cerebellum Lobules VIIb
	44	0.972	3.62	– 6	– 58	– 51	–	L Cerebellum Lobule IX
	11	0.999	3.33	– 44	12	– 38	20	L ITG
EX + CR-sham increase	–							
EX + CR-sham decrease	236	0.12	4.81	– 57	– 44	– 3	21	L MTG
	137	0.302	4.46	– 14	– 96	22	18	L SOG
	263	0.563	4.17	– 54	14	18	44	L IFG Pars Opercularis
	124	0.78	3.95	48	– 44	6	21	R MTG
	53	0.783	3.95	– 54	– 51	38	40	L IPG
	14	0.942	3.71	16	– 22	– 10	–	R Hippocampus
	64	0.951	3.69	– 36	– 34	15	48	L Rolandic Operculum
	19	0.96	3.66	54	– 20	21	48	R Rolandic Operculum
	47	0.966	3.64	– 56	4	16	48	L Precentral Gyrus
	10	0.974	3.61	– 2	– 12	8	–	L Thalamus
	18	0.976	3.6	– 18	– 75	– 57	–	L Cerebellum Lobule VIII
	14	0.976	3.6	3	– 54	16	30	R Precuneus
	26	0.981	3.58	63	– 6	18	43	R Postcentral Gyrus
	36	0.987	3.54	34	– 20	3	48	R Insula
	16	0.987	3.54	62	– 26	– 10	20	R MTG
	14	0.999	3.36	– 2	24	– 18	11	L Gyrus Rectus
CR + EX-sham increase	27	0.471	4.27	48	– 50	– 45	–	R Cerebellum Crus II
	19	0.901	3.79	46	22	– 4	–	R IFG Pars Orbitalis
	10	0.998	3.37	44	– 70	16	39	R MTG
CR + EX-sham decrease	894	0.108	4.84	28	– 28	2	–	R Hippocampus
	167	0.184	4.66	6	– 81	0	17	R Lingual Gyrus
	160	0.298	4.47	– 20	10	– 24	–	L Inferior OFC
	224	0.333	4.42	– 46	– 54	– 8	37	L ITG
	116	0.695	4.04	– 58	– 3	– 10	22	L MTG
	129	0.752	3.98	– 44	– 2	– 48	–	L ITG
	25	0.891	3.81	– 39	– 18	20	48	L Rolandic Operculum
	72	0.912	3.77	– 33	48	– 15	11	L IFG Pars Orbitalis
	89	0.92	3.76	– 50	– 14	– 8	48	L MTG
	36	0.976	3.6	22	– 64	26	18	R Cuneus
	53	0.985	3.55	– 14	56	– 18	11	L Superior OFC
	59	0.988	3.53	21	– 94	– 4	18	R IOG
	30	0.991	3.5	– 12	39	– 27	11	L Superior OFC
	13	0.992	3.49	20	8	– 16	-	R Amygdala
	11	0.993	3.48	– 63	– 16	– 6	22	L MTG
	15	0.994	3.46	30	– 75	– 4	19	R Fusiform Gyrus
	23	0.997	3.42	– 60	– 21	– 26	20	L ITG
CR + EX increase	–							
CR + EX decrease	243	0.215	4.6	8	– 62	– 34	–	R Cerebellum Lobule VIII
	229	0.545	4.19	– 40	– 72	– 39	–	L Cerebellum Crus II
	42	0.976	3.6	– 26	– 30	– 26	–	L Cerebellum Lobules IV/V
	17	0.977	3.6	63	– 9	– 28	21	R ITG
	21	0.981	3.58	– 9	– 66	– 45	–	L Cerebellum Lobule VIII
	18	0.988	3.53	– 26	– 68	– 3	19	L Lingual Gyrus
	33	0.991	3.5	– 45	– 44	– 27	37	L ITG
	16	0.999	3.34	– 15	– 40	– 44	–	L Cerebellum Lobule IX

Results analyzed at cluster extent threshold = 10 voxels, *p* < 0.001 uncorrected and *p* < 0.05 FWE-corrected

*BA* Brodmann Area, *CR* Cognitive rehabilitation, *CR-sham* Sham cognitive rehabilitation, *EX* Aerobic exercise, *EX-sham* Sham exercise, *IFG* Inferior Frontal Gyrus, *IOG* Inferior Occipital Gyrus, *IPG* Inferior Parietal Gyrus, *ITG* Inferior Temporal Gyrus, *kE* Cluster extent, *L* Left, *MTG* Middle Temporal Gyrus, *OFC* Orbitofrontal Cortex, *PHG* Parahippocampal Gyrus, *R* Right, *SMA* Supplementary Motor Area, *SMG* Supramarginal Gyrus, *SOG* Superior Occipital Gyrus

**Table 3:** Between-group longitudinal volumetric Changes at week 12 vs baseline

Comparison	kE	pFWE	F/T	MNIx	MNIy	MNIz	BA	Area
Group effect	18	0.908	8.17	8	– 54	3	10	R Lingual Gyrus
	25	0.918	8.12	– 15	– 50	– 42	–	L Cerebellum Lobule IX
	43	0.998	7.03	10	– 70	– 36	–	R Cerebellum Lobule VIII
EX + CR-sham > CR-sham + EX-sham	91	0.453	4.29	– 15	– 50	– 44	–	L Cerebellum Lobule IX
EX + CR-sham < CR-sham + EX-sham	52	0.665	4.07	66	– 14	18	22	R Postcentral Gyrus
	24	0.956	3.67	– 54	– 50	38	40	L IPG
	16	0.963	3.65	– 2	– 14	8	–	L Thalamus
	15	0.983	3.56	66	– 22	22	2	R SMG
	13	0.999	3.32	24	– 74	– 12	18	R Lingual Gyrus
CR + EX-sham > CR-sham + EX-sham	24	0.994	3.47	46	– 50	– 46	–	R Cerebellum Crus II
CR + EX-sham < CR-sham + EX-sham	60	0.199	4.63	8	– 54	3	18	R Lingual Gyrus
	346	0.73	4.01	34	– 22	– 6	–	R Hippocampus
	29	0.988	3.53	– 12	34	– 26	11	L Superior OFC
CR + EX > CR-sham + EX-sham	–							
CR + EX < CR-sham + EX-sham	76	0.817	3.91	– 26	– 28	– 27	30	L Fusiform Gyrus
	16	0.94	3.71	– 46	– 46	– 27	37	L ITG
	12	0.96	3.66	8	– 56	3	18	R Lingual Gyrus
	24	0.994	3.46	10	– 64	– 38	–	R Cerebellum Lobule VIII
CR + EX-sham > EX + CR-sham	150	0.574	4.16	-52	12	18	44	L IFG Pars Opercularis
	125	0.82	3.91	63	– 14	18	48	R Postcentral Gyrus
	90	0.972	3.62	42	– 68	15	37	R MTG
	17	0.99	3.51	– 14	– 96	22	18	L SOG
CR + EX-sham < EX + CR-sham	19	0.984	3.56	26	– 96	– 4	18	R Calcarine Sulcus
CR + EX > EX + CR-sham	15	0.896	3.8	– 54	14	18	44	L IFG Pars Opercularis
	53	0.98	3.58	– 14	– 96	24	18	L SOG
CR + EX < EX + CR-sham	312	0.308	4.46	10	– 70	– 36	–	R Cerebellum Lobule VIII
	54	0.941	3.71	– 14	– 52	– 40	–	L Cerebellum Lobule IX
	12	0.983	3.57	– 18	– 45	– 18	–	L Cerebellum Lobules IV/V
	22	0.996	3.43	– 39	– 72	– 40	–	L Cerebellum Crus II
CR + EX > CR + EX-sham	–							
CR + EX < CR + EX-sham	32	0.748	3.99	63	– 10	– 28	20	R ITG
	110	0.824	3.9	– 24	– 33	– 26	–	L Cerebellum Lobules IV/V
EX > EX-sham	–							
EX < EX-sham	93	0.44	4.3	64	– 15	18	–	R Postcentral Gyrus
	16	0.893	3.81	6	66	– 16	11	R Superior OFC
	43	0.974	3.61	– 26	– 30	– 26	–	L Cerebellum Lobules IV/V
	41	0.978	3.59	57	– 8	21	43	R Postcentral Gyrus
CR > CR-sham	61	0.974	3.61	46	– 70	33	39	R Angular Gyrus
	10	0.999	3.31	– 14	– 98	22	18	L SOG
CR < CR-sham	42	0.345	4.41	8	– 54	2	18	R Lingual Gyrus
	173	0.713	4.02	9	– 62	– 34	–	R Cerebellum Lobule VIII
	18	0.987	3.54	– 20	– 48	– 16	–	L Cerebellum Lobules IV/V

Results analyzed at cluster extent threshold = 10 voxels, *p* < 0.001 uncorrected and *p* < 0.05 FWE-corrected

*BA* Brodmann Area, *CR* Cognitive rehabilitation, *CR-sham* Sham cognitive rehabilitation, *EX* Aerobic exercise, *EX-sham* Sham exercise, *IFG* Inferior Frontal Gyrus, *IPG* Inferior Parietal Gyrus, *ITG* Inferior Temporal Gyrus, *kE* Cluster extent, *L* Left, *MTG* Middle Temporal Gyrus, *OFC* Orbitofrontal Cortex, *R* Right, *SMG* Supramarginal Gyrus, *SOG* Superior Occipital Gyrus

There were no significant correlations between longitudinal volumetric changes from baseline to W12 and changes in cognitive performances (FWE-corrected threshold). Correlations significant at the uncorrected threshold are reported in Supplementary Table 1.

**Fig. 1:**
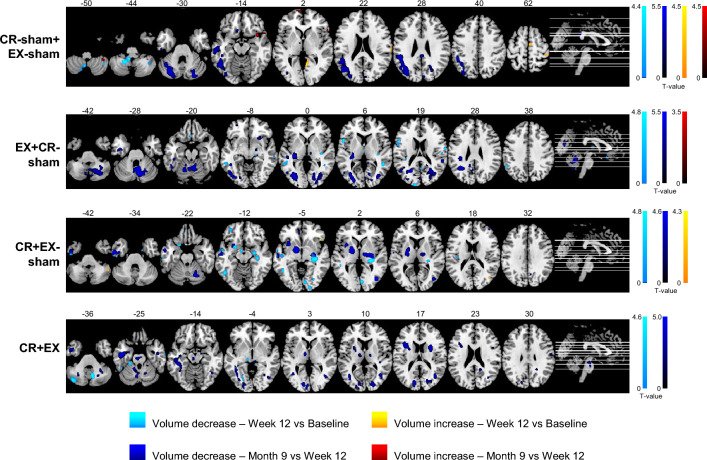
Within-group volume decreases and increases from baseline to W12 and from W12 to M9 in the four treatment groups (*p* < 0.001, uncorrected, cluster extent threshold = 10 voxels). Significant clusters at the two time points were overlaid on the ch2better template in MRIcron (https://www.nitrc.org/projects/mricron) and axial slices, with the corresponding MNI z coordinate shown on top, were extracted. Images are presented in neurological convention

Longitudinal within-group volumetric changes from baseline to W12 and from W12 to M9 are shown in Fig. 1.

#### Longitudinal differences – M9 vs W12

Within-group volumetric changes from W12 to M9 showed several clusters of decreased volume in occipital, temporal, cerebellar and subcortical areas in all groups (FWE-corrected and uncorrected thresholds).

Longitudinal volumetric changes from W12 to M9 were not significantly different between the four treatment arms (FWE-corrected threshold).

At the uncorrected threshold, there was no significant effect of treatment.

Detailed findings from this analysis are reported in Tables 4 and 5.

**Table 4:** Within-group longitudinal volumetric Changes at month 9 vs week 12

Comparison	kE	pFWE	T	MNIx	MNIy	MNIz	BA	Area
CR-sham + EX-sham increase	13	0.225	4.51	16	6	– 32	28	R PHG
	44	0.38	4.29	51	20	– 16	38	R STP
	44	0.485	4.18	-8	69	3	10	L Medial SFG
	58	0.493	4.17	36	14	– 16	38	R Insula
	19	0.677	3.98	56	24	0	45	R IFG Pars Triangularis
	35	0.722	3.94	38	– 44	– 50	–	R Cerebellum Lobule VIII
	10	0.794	3.86	52	14	– 3	–	R Insula
CR-sham + EX-sham decrease	1032	0.011	5.51	– 30	– 72	– 27	–	L Cerebellum Crus I
	5416	0.112	4.76	– 44	– 69	18	39	L MOG
	406	0.227	4.51	27	– 82	– 32	-	R Cerebellum Crus I
	475	0.331	4.35	– 57	– 21	– 10	20	L MTG
	34	0.52	4.14	15	– 75	14	18	R Calcarine Sulcus
	350	0.522	4.14	24	– 69	32	19	R SOG
	241	0.617	4.05	15	– 90	– 4	18	R Lingual Gyrus
	70	0.741	3.92	0	– 36	27	23	R PCC
	36	0.787	3.87	– 9	-8	6	–	L Thalamus
	60	0.895	3.72	– 34	16	12	48	L Insula
	70	0.921	3.67	– 54	– 3	– 9	22	L STG
	46	0.93	3.65	4	– 84	26	18	R Cuneus
	20	0.958	3.58	– 45	– 34	– 15	20	L ITG
	60	0.963	3.57	– 9	24	– 16	11	L Gyrus Rectus
	23	0.967	3.55	– 51	– 21	– 32	20	L ITG
	20	0.977	3.51	34	– 81	10	19	R MOG
	26	0.982	3.48	16	– 74	54	7	R SPG
	13	0.983	3.48	24	– 84	34	19	R SOG
	21	0.988	3.44	16	– 84	15	19	R Calcarine Sulcus
	18	0.993	3.4	9	– 56	14	30	R Calcarine Sulcus
EX + CR-sham increase	11	0.979	3.5	66	– 4	20	43	R Postcentral Gyrus
EX + CR-sham decrease	3287	0.012	5.48	4	– 56	– 26	–	Cerebellar Vermis VIII
	419	0.015	5.4	– 32	– 28	2	–	L Hippocampus
	3588	0.021	5.3	– 36	– 72	0	19	L MOG
	194	0.197	4.56	– 16	– 62	– 18	–	L Cerebellum Lobule VI
	1679	0.327	4.36	24	– 58	16	17	R Calcarine Sulcus
	115	0.372	4.3	56	– 38	18	42	R STG
	152	0.551	4.11	3	– 82	12	18	L Calcarine Sulcus
	215	0.652	4.01	– 45	– 12	– 27	20	L ITG
	93	0.733	3.93	– 8	– 69	– 44	–	L Cerebellum Lobule VIII
	139	0.76	3.9	26	18	– 10	48	R Insula
	50	0.875	3.75	– 30	51	– 10	11	L Middle OFC
	185	0.904	3.7	– 14	– 50	30	–	L Precuneus
	25	0.936	3.64	16	– 48	21	–	R Precuneus
	51	0.945	3.62	– 50	– 27	– 2	21	L MTG
	41	0.955	3.59	– 22	– 100	4	17	L MOG
	28	0.96	3.58	9	-42	9	29	R Precuneus
	30	0.972	3.53	28	56	– 12	11	R Middle OFC
	30	0.978	3.51	– 15	– 46	– 21	–	L Cerebellum Lobules IV/V
	19	0.985	3.47	10	– 64	– 58	–	R Cerebellum Lobule VIII
	14	0.99	3.43	– 62	– 51	10	21	L MTG
	17	0.996	3.35	– 20	– 76	24	19	L SOG
	11	0.996	3.35	– 14	– 72	18	18	L Calcarine Sulcus
	15	0.998	3.28	– 24	– 60	– 33	–	L Cerebellum Lobule VI
CR + EX-sham increase	–							
CR + EX-sham decrease	1062	0.193	4.57	– 9	– 2	– 6	–	L Pallidum
	924	0.231	4.5	– 52	– 8	– 34	20	L ITG
	188	0.241	4.48	42	– 74	3	19	R MOG
	687	0.313	4.38	32	– 14	– 2	-	R Putamen
	366	0.7	3.96	– 33	12	0	48	L Insula
	30	0.785	3.87	8	– 63	36	7	R Precuneus
	235	0.808	3.84	44	39	27	45	R IFG Pars Triangularis
	235	0.847	3.79	24	– 68	– 26	–	R Cerebellum Lobule VI
	41	0.936	3.64	– 27	– 72	– 9	18	L Fusiform Gyrus
	23	0.977	3.51	– 6	39	– 6	11	L ACC
	10	0.98	3.5	– 58	– 9	8	22	L STG
	23	0.991	3.42	58	– 9	– 18	21	R MTG
CR + EX increase	–							
CR + EX decrease	2349	0.051	5.02	– 45	– 14	– 24	20	L ITG
	744	0.288	4.41	– 15	– 86	4	17	L SOG
	540	0.387	4.29	39	– 64	16	39	R MTG
	165	0.573	4.09	– 10	– 9	6	–	L Thalamus
	451	0.605	4.06	18	– 84	9	18	R Calcarine Sulcus
	611	0.698	3.96	– 39	15	18	48	L IFG Pars Opercularis
	150	0.744	3.91	45	– 6	– 30	20	R ITG
	327	0.812	3.84	– 6	– 66	– 27	–	L Cerebellum Lobule VI
	148	0.856	3.78	– 24	– 52	– 30	–	L Cerebellum Lobule VIII
	104	0.877	3.75	15	– 50	24	23	R Precuneus
	142	0.899	3.71	34	– 58	– 32	–	R Cerebellum Crus I
	210	0.927	3.66	9	– 56	– 32	–	R Cerebellum Lobule VIII
	43	0.938	3.64	– 16	– 81	33	18	L SOG
	35	0.954	3.6	– 52	– 51	40	40	L IPG
	59	0.967	3.55	21	– 38	– 28	–	R Cerebellum Lobules I/IV
	32	0.973	3.53	57	– 10	32	43	R Postcentral Gyrus
	11	0.986	3.46	– 22	– 62	0	19	L Lingual Gyrus
	20	0.987	3.45	14	– 12	9	–	R Thalamus
	23	0.988	3.45	46	6	26	44	R IFG Pars Opercularis
	14	0.988	3.45	15	– 9	– 14	–	R Hippocampus
	20	0.989	3.44	– 30	– 51	– 44	–	L Cerebellum Lobule VIII
	36	0.991	3.42	44	– 57	– 15	37	R ITG
	10	0.996	3.35	30	– 63	– 3	19	R Fusiform Gyrus

Results analyzed at cluster extent threshold = 10 voxels, *p* < 0.001 uncorrected and *p* < 0.05 FWE-corrected

*ACC* Anterior Cingulate Cortex, *BA* Brodmann Area, *CR* Cognitive rehabilitation, *CR-sham* Sham cognitive rehabilitation, *EX* Aerobic exercise, *EX-sham* Sham exercise, *IFG* Inferior Frontal Gyrus, *IOG* Inferior Occipital Gyrus, *IPG* Inferior Parietal Gyrus, *ITG* Inferior Temporal Gyrus, *kE* Cluster extent, *L* Left, *MFG* Middle Frontal Gyrus, *MOG* Middle Occipital Gyrus, *MTG* Middle Temporal Gyrus, *OFC* Orbitofrontal Cortex, *PCC* Posterior Cingulate Cortex, *PHG* Parahippocampal Gyrus, *R* Right, *SFG* Superior Frontal Gyrus, *SOG* Superior Occipital Gyrus, *SPG* Superior Parietal Gyrus, *STG* Superior Temporal Gyrus, *STP* Superior Temporal Pole

**Table 5:** Between-group longitudinal volumetric Changes at month 9 vs week 12

Comparison	kE	pFWE	T	MNIx	MNIy	MNIz	BA	Area
EX + CR-sham > CR-sham + EX-sham	10	0.995	3.36	– 10	22	– 21	11	L Gyrus Rectus
EX + CR-sham < CR-sham + EX-sham	60	0.596	4.07	56	– 36	20	42	R STG
	22	0.953	3.6	– 8	– 68	-46	–	L Cerebellum Lobule VIII
	10	0.988	3.45	6	– 58	– 36	–	Cerebellar Vermis IX
	22	0.993	3.4	30	57	– 12	11	R Middle OFC
	27	0.994	3.39	33	– 51	– 48	–	R Cerebellum Lobule VIII
CR + EX-sham > CR-sham + EX-sham	–							
CR + EX-sham < CR-sham + EX-sham	43	0.84	3.8	38	16	– 14	38	R Insula
	42	0.859	3.78	52	24	30	44	R IFG Pars Triangularis
	15	0.997	3.32	39	– 63	– 54	–	R Cerebellum Lobule VIII
CR + EX > CR-sham + EX-sham	28	0.833	3.81	– 58	– 3	0	48	L STG
CR + EX < CR-sham + EX-sham	71	0.897	3.72	56	– 9	28	43	R Postcentral Gyrus
	10	0.994	3.38	38	– 58	– 58	–	R Cerebellum Lobule VIII
CR + EX-sham > EX + CR-sham	204	0.343	4.34	8	– 74	– 30	–	R Cerebellum Crus II
	33	0.978	3.51	– 51	– 66	8	37	L MTG
CR + EX-sham < EX + CR-sham	38	0.941	3.63	39	40	34	46	R MFG
	12	0.986	3.46	– 52	12	– 22	38	L MTG
CR + EX > EX + CR-sham	–							
CR + EX < EX + CR-sham	–							
CR + EX > CR + EX-sham	101	0.701	3.96	– 38	6	0	48	L Insula
	19	0.895	3.72	– 58	– 8	6	48	L STG
	18	0.978	3.51	42	51	16	46	R MFG
	25	0.988	3.45	– 60	– 18	– 8	21	L MTG
CR + EX < CR + EX-sham	–							
EX > EX-sham	18	0.955	3.59	– 45	21	– 4	47	L IFG Pars Orbitalis
EX < EX-sham	261	0.503	4.16	30	– 48	– 42	–	R Cerebellum Lobule VIII
	33	0.892	3.73	– 8	– 68	– 46	–	L Cerebellum Lobule VIII
	39	0.944	3.62	– 34	– 28	0	–	L Hippocampus
	25	0.967	3.55	15	32	– 14	11	R Superior OFC
	33	0.969	3.54	12	50	– 16	11	R Gyrus Rectus
	17	0.982	3.49	9	– 57	– 33	–	R Cerebellum Lobule VIII
	12	0.994	3.38	8	– 74	– 28	–	R Cerebellum Crus II
CR > CR-sham	49	0.967	3.55	– 46	– 75	– 16	19	L IOG
	30	0.988	3.44	– 51	– 60	– 18	37	L ITG
CR < CR-sham	10	0.956	3.59	52	26	28	45	R IFG Pars Triangularis
	16	0.993	3.4	51	9	26	44	R IFG Pars Opercularis

Results analyzed at cluster extent threshold = 10 voxels, *p* < 0.001 uncorrected and *p* < 0.05 FWE-corrected

*BA* Brodmann Area, *CR* Cognitive rehabilitation, *CR-sham* Sham cognitive rehabilitation, *EX* Aerobic exercise, *EX-sham* Sham exercise, *IFG* Inferior Frontal Gyrus, *IOG* Inferior Occipital Gyrus, *ITG* Inferior Temporal Gyrus, *kE* Cluster extent, *L* Left, *MFG* Middle Frontal Gyrus, *MTG* Middle Temporal Gyrus, *OFC* Orbitofrontal Cortex, *R* Right, *STG* Superior Temporal Gyrus

There were no significant correlations between longitudinal volumetric changes from W12 to M9 and changes in cognitive performances (FWE-corrected threshold). Correlations significant at the uncorrected threshold are reported in Supplementary Table 2.

#### Longitudinal differences – M9 changes vs W12 changes

**Fig. 2:**
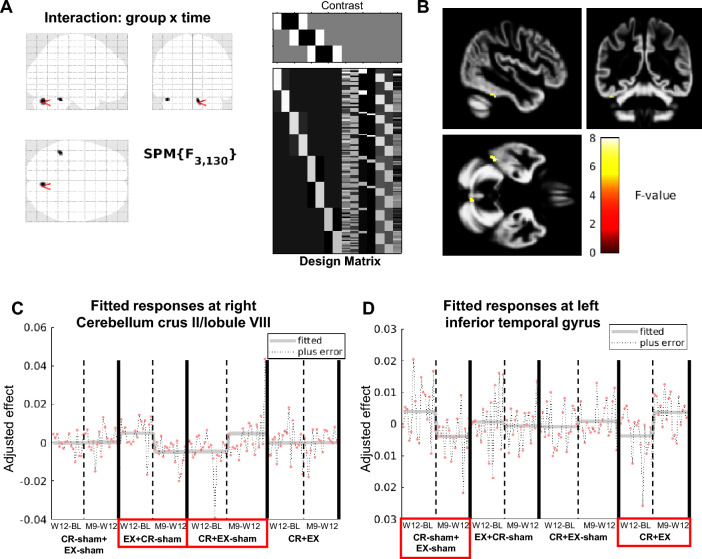
Volumetric longitudinal changes in the four treatment groups: comparison between changes at M9 vs W12 and changes at W12 vs baseline (*p* < 0.001, uncorrected, cluster extent threshold = 10 voxels). **A** Significant clusters are shown on the left side, projected onto a glass brain. The design matrix shown on the right side contains eight cells representing longitudinal changes in the four groups at W12 vs baseline (W12-BL) and at M9 vs W12 (M9-W12), and the seven additional covariates (age, sex, four dummy variables for the acquisition centers, and follow-up length). Above the design matrix, the structure of the F-contrast (group-by-time interaction, as computed in SPM12) is reported. **B** Significant clusters overlaid on the customised grey matter template image. **C** Demeaned and adjusted group effects at peak-level of the significant cluster in Cerebellum crus II/lobule VIII are plotted (grey line). The different behavior between EX + CR-sham and CR + EX-sham can be observed. **D** The same plot for the significant cluster in the inferior temporal gyrus is shown. Here, the different behavior between CR-sham + EX-sham and CR + EX can be observed

There was a significant difference in longitudinal volumetric changes at M9 vs W12 and changes at W12 vs baseline between the four treatment arms (FWE-corrected threshold and significant group-by-time interaction). In particular, there were significantly different patterns of change in left inferior temporal gyrus volume between CR + EX and CR-sham + EX-sham, where volume decreased from baseline to W12 and was stable from W12 to M9 in CR + EX, and was stable from baseline to W12 and decreased from W12 to M9 in CR-sham + EX-sham. Also, there were significantly different patterns of change in right cerebellum crus II volume between EX + CR-sham and CR + EX-sham, which was stable from baseline to W12 in EX + CR-sham and decreased from W12 to M9 in CR + EX-sham, where volume decreased at W12 and was stable at M9. Figure 2 shows results of the group-by-time interaction as assessed with SPM12.

Detailed findings from this analysis are reported in Table 6.

**Table 6:** Between-group longitudinal volumetric differences in the Changes at month 9 vs week 12 and Changes at week 12 vs baseline

Comparison	kE	pFWE	F/T	MNIx	MNIy	MNIz	BA	Area
Group by time interaction	93	0.705	7.97	8	– 74	– 30	–	R Cerebellum Crus II
	29	0.762	7.82	– 45	– 45	– 27	–	L ITG
EX + CR-sham > CR-sham + EX-sham	74	0.943	3.54	– 16	– 52	– 42	–	L Cerebellum Lobule IX
	19	0.987	3.39	6	– 72	– 42	–	R Cerebellum Lobule VIIb
	20	0.996	3.29	34	– 54	– 48	–	R Cerebellum Lobule VIII
EX + CR-sham < CR-sham + EX-sham	32	0.716	3.83	– 52	34	– 6	–	L IFG Pars Orbitalis
	36	0.789	3.76	22	– 90	32	18	R SOG
	99	0.932	3.57	– 4	26	– 18	11	L Gyrus Rectus
	42	0.966	3.48	– 50	16	3	45	L IFG Pars Triangularis
	28	0.971	3.47	12	– 82	– 14	–	R Cerebellum Lobule VI
	23	0.976	3.44	64	– 4	18	43	R Postcentral Gyrus
	14	0.994	3.32	– 2	– 12	6	–	L Thalamus
CR + EX-sham > CR-sham + EX-sham	45	0.846	3.7	48	– 75	6	19	R MOG
	43	0.945	3.54	40	– 62	– 54	–	R Cerebellum Lobule VIIb
	32	0.972	3.46	– 33	– 63	– 62	–	L Cerebellum Lobule VIII
CR + EX-sham < CR-sham + EX-sham	728	0.422	4.1	– 54	– 60	– 20	37	L ITG
	21	0.481	4.05	– 24	60	– 15	11	L Middle OFC
	120	0.774	3.78	– 34	– 64	32	19	L MOG
	38	0.9	3.62	– 3	– 15	8	–	L Thalamus
	36	0.991	3.36	– 28	– 87	– 40	–	L Cerebellum Crus II
CR + EX > CR-sham + EX-sham	72	0.734	3.82	38	– 48	– 48	–	R Cerebellum Lobule VIII
CR + EX < CR-sham + EX-sham	75	0.053	4.78	– 44	– 42	– 28	37	L ITG
	11	0.965	3.49	– 20	– 22	– 26	–	L PHG
	10	0.997	3.28	– 36	– 76	– 39	–	L Cerebellum Crus II
CR + EX-sham > EX + CR-sham	67	0.952	3.52	-52	10	8	48	L IFG Pars Opercularis
	32	0.97	3.47	64	– 4	18	43	R Postcentral Gyrus
	19	0.976	3.45	46	– 75	0	19	R IOG
	32	0.989	3.37	45	– 70	33	39	R Angular Gyrus
CR + EX-sham < EX + CR-sham	407	0.04	4.86	8	– 72	– 30	–	R Cerebellum Crus II
	23	0.979	3.43	– 46	– 75	– 15	19	L IOG
	18	0.989	3.37	– 14	– 60	– 40	–	L Cerebellum Lobule VIII
	28	0.996	3.29	– 50	– 66	– 8	37	L ITG
CR + EX > EX + CR-sham	13	0.996	3.3	44	– 69	33	39	R Angular Gyrus
CR + EX < EX + CR-sham	15	0.922	3.59	– 6	– 34	– 15	–	L Cerebellum Lobule III
	82	0.937	3.56	8	– 60	– 36	–	R Cerebellum Lobule VIII
	10	0.998	3.26	– 36	– 75	– 40	–	L Cerebellum Crus II
CR + EX > CR + EX-sham	–							
CR + EX < CR + EX-sham	–							
EX > EX-sham	46	0.872	3.66	6	– 74	– 27	–	Cerebellar Vermis VII
	15	0.996	3.29	34	– 50	– 46	–	R Cerebellum Lobule VIII
EX < EX-sham	21	0.906	3.61	66	– 16	20	48	R SMG
	28	0.987	3.39	– 14	21	– 18	11	L Superior OFC
	17	0.995	3.31	– 56	30	4	45	L IFG Pars Triangularis
CR > CR-sham	30	0.969	3.47	48	– 74	6	19	R MOG
	42	0.971	3.46	45	– 69	32	39	R Angular Gyrus
CR < CR-sham	100	0.47	4.06	– 44	– 44	– 27	–	L ITG
	52	0.589	3.95	8	– 94	– 9	–	R Lingual Gyrus
	124	0.641	3.9	– 20	– 45	– 16	–	L Cerebellum Lobules IV/V
	87	0.813	3.74	– 46	– 74	– 15	19	L IOG
	113	0.831	3.72	9	– 70	– 33	–	R Cerebellum Lobule VIII
	62	0.962	3.5	– 45	– 70	– 42	–	L Cerebellum Crus II

Results analyzed at cluster extent threshold = 10 voxels, *p* < 0.001 uncorrected and *p* < 0.05 FWE-corrected

*BA* Brodmann Area, *CR* Cognitive rehabilitation, *CR-sham* Sham cognitive rehabilitation, *EX* Aerobic exercise, *EX-sham* Sham exercise, *IFG* Inferior Frontal Gyrus, *IOG* Inferior Occipital Gyrus, *ITG* Inferior Temporal Gyrus, *kE* Cluster extent, *L* Left, *MOG* Middle Occipital Gyrus, *OFC* Orbitofrontal Cortex, *PHG* Parahippocampal Gyrus, *R* Right, *SMG* Supramarginal Gyrus, *SOG* Superior Occipital Gyrus

### Diffusivity analysis

There were no within-group longitudinal changes and no between-group differences in WM FA in any of the four treatment arms (FWE-corrected and uncorrected thresholds).

## Discussion

The analysis of volumetric modifications after treatment did not highlight clear effects of the interventions. In fact, we found a mixed pattern of volume increase and decrease in several areas in all groups. In general, only a few differences between groups were found, all not surviving the corrected threshold and only partially specific to a single group or intervention. In comparison, the effect of the disease, characterized by volume reductions in several areas (Fig. 1), was evident in all groups. This was even more pronounced in data collected 6 months after treatment, where intervention effects were extremely limited, showing only a small number of sub-threshold differences between groups, while significant progression of atrophy, representing the natural course of structural damage characterizing PMS patients [29], was present in all groups.

These observations led us to hypothesize that, due to the effect of MS-related atrophy, our initial analysis might not have been able to highlight subtler differences between interventions. To uncover possible treatment effects that previously went unnoticed, we contrasted changes observed after treatment to those seen after follow-up/observation, as there are studies showing changes in the first week of training, which tend to disappear two months after the termination of training [30]. We reasoned that, given the small number of patients included in each group, there could be high variability between groups, which could be mitigated by assessing intervention effects as a difference in the rate of volumetric changes between the two periods. This analysis also made it possible to differentiate immediate from delayed effects of the intervention and to characterize whether observed differences were due to enlargement of brain areas or a reduction in volume loss (i.e., neuroprotective effect).

The results of this analysis showed significantly different rates of volume change between the four treatment groups in the right cerebellum crus II and in the left inferior temporal cortex. In particular, left inferior temporal gyrus volume was stable during treatment and decreased after the follow-up in CR-sham + EX-sham, while the opposite pattern was observed in CR + EX, where it decreased after treatment and remained stable at follow-up. The inferior temporal gyrus, along with other structures of this lobe, is involved in semantic memory [31, 32] and changes in this region were also reported in a trial involving patients with MS, where five weeks of CR increased its functional activity during the performance of a memory task [33]. Regarding right cerebellum crus II volume, we found that it remained stable after treatment and decreased at follow-up in EX + CR-sham, while the opposite pattern of change was observed in CR + EX-sham, where volume in this region decreased during treatment and was stable at follow-up. This result might be tied to the repetitive stepping motion performed during aerobic exercise in the EX + CR-sham group, in fact this area has been demonstrated to contribute to the accurate temporal prediction of absolute timing, which is linked to the controlled repetition of a motor action [34]. Additionally, this region is part of the second non-motor representation of the cerebellum and has been shown to be involved in cognitive, emotional and social tasks [35], taking part in both language processing and working memory. Considering that both this difference and the change observed in the inferior temporal gyrus were significant in the comparison between groups that performed CR versus CR-sham, it could be possible that the CR component of the intervention is the primary driver of these modifications. However, the absence of significant correlations with improvements in cognitive functions makes it difficult to ascribe such meaning to our findings. Indeed, they could also be explained by heterogeneous atrophy dynamics between patients and a limited number of participants per group included.

We observed no effects of either CR, EX or their combination on DTI measures of WM integrity. Previous studies on this topic have found mixed results, however most included patients with relatively low disability [36]. The only study involving patients with PMS and high disability found no effects of aerobic training on WM microstructure, measured with graph metrics of structural connectivity [37]. This might indicate that WM structural plasticity in these patients is severely limited, possibly due to a depletion of their reserve after many years of disease. It is also possible that 12 weeks of training were not sufficient to impact WM microstructure, and longer treatment durations might show different results. However, longitudinal assessments in people with MS have highlighted a decline in measures of WM integrity over time, depending on the duration of follow-up and on the methodology employed for data analysis [38–40]. In the present study, the stability of FA values found in contrast to the progression of atrophy may also suggest a stabilizing effect of the administered training, similarly to the neuroprotective effects observed after treatment with some pharmacological therapies [41, 42]. Lastly, the method applied for this analysis has been optimized to improve the quality of the superposition between anatomically corresponding fiber bundles, in order to facilitate the possibility of detecting training-induced changes. Nonetheless, we cannot exclude the fact that some changes could have happened but not exactly at the same voxel level in all participants, and thus they might not have been captured in our analysis.

Compared with the results reported in the previously published analysis of global brain volumes and task-related fMRI activity from the CogEx MRI substudy [8]—where patients who underwent CR exhibited increased cortical GM volume after 12 weeks of training—the findings of the present study do not reveal substantial volume increases. One possible explanation for this difference is that the global volume increase observed previously may not correspond to superimposable local variations at the voxel-based level, considering also that the current analysis was not confined to specific tissues or regions, but assessed the whole brain. In fact, the findings from the previous study could suggest that global and lobar increases in cortical GM volume outweighed the more widespread trend of general decrease, resulting in a net positive effect. In addition, it is important to consider that the previous study included 84 patients in the analysis of W12 data (see study flowchart [8]), of which 12 had to be excluded from the current volumetric analysis, given that only patients having complete assessments at all three time points were suitable for TBM. These exclusions additionally resulted in a baseline imbalance between groups in CVLT and, more importantly, NBV, which could have further contributed to the observed discrepancies between the findings of the two studies. Considering the results of the main CogEx trial [5], the fact that the improvement observed in the main outcome was not different between the four treatment groups might indicate that there are no specific neural substrates underlying these changes. However, similar behaviors between the two groups of patients who underwent CR were observed in both of the MRI analyses performed so far, so there might be common mechanisms at play.

There are some limitations to this work. Despite the robustness of the methodology for MRI data analysis, the small number of patients with a complete assessment in each treatment group could have introduced a high degree of variability in the longitudinal changes observed at voxel level. Also, considering the extensive damage and limited capacity for structural improvements due to a depletion of brain reserves typically observed in PMS patients [7], performing an MRI scan before the baseline visit would have given us a reference to assess disease effects on neurodegeneration in each patient and to better disentangle the effects of the intervention. Lastly, findings on structural adaptations after rehabilitation in MS are still quite heterogeneous, as evidenced by a recent review [36]. While 12 weeks of treatment might be deemed sufficient in this context, based on results in healthy aging subjects [17, 18], longer treatment durations or higher intensities of training might be needed to observe more consistent effects in PMS patients, also considering the limitations outlined above.

In conclusion, the included cohort of cognitively impaired patients with PMS displayed no differences between treatment groups in localized volumetric or diffusivity changes. A trend of volume decrease in several cortical regions, likely following the natural trajectory of PMS-related neurodegeneration, was observed in all groups over the trial period. In contrast, WM FA remained generally stable, indicative of a possible neuroprotective effect. We can hypothesize that CR combined with either EX or EX-sham might result in volumetric changes of areas relevant for cognitive functions, while EX might support structural changes in motor-related cerebellar regions. However, due to the absence of relevant correlations with cognitive performance improvements other works are needed to confirm these findings.

## Supplementary Information

Below is the link to the electronic supplementary material.

Supplementary file1 (DOCX 28 KB)

Supplementary file1 (DOCX 21 KB)

## Data Availability

Anonymized data are available one year after publication, upon reasonable request. Please make the request to the corresponding author. A CogEx Committee will review the request for approval. A data sharing agreement will be produced before any data is shared.

## References

[1] Chiaravalloti ND, DeLuca J (2008) Cognitive impairment in multiple sclerosis. Lancet Neurol 7(12):1139–1151 19007738 10.1016/S1474-4422(08)70259-X

[2] Mistri D et al (2024) Cognitive phenotypes in multiple sclerosis: mapping the spectrum of impairment. J Neurol 271(4):1571–1583 38007408 10.1007/s00415-023-12102-5

[3] De Meo E et al (2021) Identifying the distinct cognitive phenotypes in multiple sclerosis. JAMA Neurol 78(4):414–425 33393981 10.1001/jamaneurol.2020.4920 PMC7783596

[4] Zackowski KM et al (2021) Prioritizing progressive MS rehabilitation research: a call from the International Progressive MS Alliance. Mult Scler 27(7):989–1001 33720795 10.1177/1352458521999970 PMC8151585

[5] Feinstein A et al (2023) Cognitive rehabilitation and aerobic exercise for cognitive impairment in people with progressive multiple sclerosis (CogEx): a randomised, blinded, sham-controlled trial. Lancet Neurol 22(10):912–924 37739574 10.1016/S1474-4422(23)00280-6

[6] Brandstadter R, Sand IK, Sumowski JF (2019) Beyond rehabilitation: a prevention model of reserve and brain maintenance in multiple sclerosis. Mult Scler J 25(10):1372–1378 10.1177/1352458519856847 PMC6719722 31469354

[7] Vollmer TL et al (2021) Multiple sclerosis phenotypes as a continuum the role of neurologic reserve. Neurology-Clinical Practice 11(4):342–351 34476126 10.1212/CPJ.0000000000001045 PMC8382415

[8] Rocca MA et al (2024) Cognitive rehabilitation effects on grey matter volume and Go-NoGo activity in progressive multiple sclerosis: results from the CogEx trial. J Neurol Neurosurg Psychiatry. 10.1136/jnnp-2024-333460 38754979 10.1136/jnnp-2024-333460

[9] Bodini B et al (2009) Exploring the relationship between white matter and gray matter damage in early primary progressive multiple sclerosis: an in vivo study with TBSS and VBM. Hum Brain Mapp 30(9):2852–2861 19172648 10.1002/hbm.20713 PMC6871131

[10] Henry RG et al (2008) Regional grey matter atrophy in clinically isolated syndromes at presentation. J Neurol Neurosurg Psychiatry 79(11):1236–1244 18469033 10.1136/jnnp.2007.134825 PMC4827711

[11] Raz E et al (2010) Clinically isolated syndrome suggestive of multiple sclerosis: voxelwise regional investigation of white and gray matter. Radiology 254(1):227–234 20019140 10.1148/radiol.2541090817

[12] Ceccarelli A et al (2008) A voxel-based morphometry study of grey matter loss in MS patients with different clinical phenotypes. Neuroimage 42(1):315–322 18501636 10.1016/j.neuroimage.2008.04.173

[13] Filippi M et al (2012) Multiple sclerosis: effects of cognitive rehabilitation on structural and functional MR imaging measures–an explorative study. Radiology 262(3):932–940 22357892 10.1148/radiol.11111299

[14] Rocca MA et al (2019) Functional and structural plasticity following action observation training in multiple sclerosis. Mult Scler 25(11):1472–1487 30084706 10.1177/1352458518792771

[15] Prosperini L, Di Filippo M (2019) Beyond clinical changes: rehabilitation-induced neuroplasticity in MS. Mult Scler 25(10):1348–1362 31469359 10.1177/1352458519846096

[16] Zatorre RJ, Fields RD, Johansen-Berg H (2012) Plasticity in gray and white: neuroimaging changes in brain structure during learning. Nat Neurosci 15(4):528–536 22426254 10.1038/nn.3045 PMC3660656

[17] Castells-Sanchez A et al (2022) Molecular and brain volume changes following aerobic exercise, cognitive and combined training in physically inactive healthy late-middle-aged adults: the Projecte Moviment randomized controlled trial. Front Hum Neurosci 16:854175 35529777 10.3389/fnhum.2022.854175 PMC9067321

[18] Roig-Coll F et al (2024) Changes in cardiovascular health and white matter integrity with aerobic exercise, cognitive and combined training in physically inactive healthy late-middle-aged adults: the “Projecte Moviment” randomized controlled trial. Eur J Appl Physiol 124(3):909–924 37768344 10.1007/s00421-023-05319-9 PMC10879245

[19] Feinstein A et al (2020) Study protocol: improving cognition in people with progressive multiple sclerosis: a multi-arm, randomized, blinded, sham-controlled trial of cognitive rehabilitation and aerobic exercise (COGEx). BMC Neurol 20(1):204 32443981 10.1186/s12883-020-01772-7 PMC7245035

[20] Ashburner J, Ridgway GR (2012) Symmetric diffeomorphic modeling of longitudinal structural MRI. Front Neurosci 6:197 23386806 10.3389/fnins.2012.00197 PMC3564017

[21] Ashburner J (2007) A fast diffeomorphic image registration algorithm. Neuroimage 38(1):95–113 17761438 10.1016/j.neuroimage.2007.07.007

[22] Andersson JLR et al (2017) Towards a comprehensive framework for movement and distortion correction of diffusion MR images: within volume movement. Neuroimage 152:450–466 28284799 10.1016/j.neuroimage.2017.02.085 PMC5445723

[23] Basser PJ, Mattiello J, LeBihan D (1994) Estimation of the effective self-diffusion tensor from the NMR spin echo. J Magn Reson B 103(3):247–254 8019776 10.1006/jmrb.1994.1037

[24] Keihaninejad S et al (2013) An unbiased longitudinal analysis framework for tracking white matter changes using diffusion tensor imaging with application to Alzheimer’s disease. Neuroimage 72:153–163 23370057 10.1016/j.neuroimage.2013.01.044

[25] Zhang H et al (2007) High-dimensional spatial normalization of diffusion tensor images improves the detection of white matter differences: an example study using amyotrophic lateral sclerosis. IEEE Trans Med Imaging 26(11):1585–1597 18041273 10.1109/TMI.2007.906784

[26] Zhang H et al (2007) Unbiased white matter atlas construction using diffusion tensor images. Med Image Comput Comput Assist Interv 10(Pt 2):211–218 18044571 10.1007/978-3-540-75759-7_26

[27] Smith SM et al (2006) Tract-based spatial statistics: voxelwise analysis of multi-subject diffusion data. Neuroimage 31(4):1487–1505 16624579 10.1016/j.neuroimage.2006.02.024

[28] Langdon DW et al (2012) Recommendations for a brief international cognitive assessment for multiple sclerosis (BICAMS). Mult Scler 18(6):891–898 22190573 10.1177/1352458511431076 PMC3546642

[29] Rocca MA et al (2021) Association of gray matter atrophy patterns with clinical phenotype and progression in multiple sclerosis. Neurology 96(11):e1561–e1573 33441452 10.1212/WNL.0000000000011494

[30] Driemeyer J et al (2008) Changes in gray matter induced by learning-revisited. PLoS ONE. 10.1371/journal.pone.0002669 18648501 10.1371/journal.pone.0002669 PMC2447176

[31] Mummery CJ et al (2000) A voxel-based morphometry study of semantic dementia: relationship between temporal lobe atrophy and semantic memory. Ann Neurol 47(1):36–45 10632099

[32] Chan D et al (2001) Patterns of temporal lobe atrophy in semantic dementia and Alzheimer’s disease. Ann Neurol 49(4):433–442 11310620

[33] Chiaravalloti ND et al (2012) Increased cerebral activation after behavioral treatment for memory deficits in MS. J Neurol 259(7):1337–1346 22237819 10.1007/s00415-011-6353-x

[34] Yamaguchi K, Sakurai Y (2016) Inactivation of cerebellar cortical crus II disrupts temporal processing of absolute timing but not relative timing in voluntary movements. Front Syst Neurosci. 10.3389/fnsys.2016.00016 26941621 10.3389/fnsys.2016.00016 PMC4764692

[35] Guell X, Gabrieli JDE, Schmahmann JD (2018) Triple representation of language, working memory, social and emotion processing in the cerebellum: convergent evidence from task and seed-based resting-state fMRI analyses in a single large cohort. Neuroimage 172:437–449 29408539 10.1016/j.neuroimage.2018.01.082 PMC5910233

[36] Rocca MA et al (2024) Advanced neuroimaging techniques to explore the effects of motor and cognitive rehabilitation in multiple sclerosis. J Neurol 271(7):3806–3848 38691168 10.1007/s00415-024-12395-0

[37] Tilsley P et al (2023) Physical fitness moderates the association between brain network impairment and both motor function and cognition in progressive multiple sclerosis. J Neurol 270(10):4876–4888 37341806 10.1007/s00415-023-11806-y

[38] Schneider R et al (2019) Temporal dynamics of diffusion metrics in early multiple sclerosis and clinically isolated syndrome: a 2-year follow-up tract-based spatial statistics study. Front Neurol 10:1165 31749760 10.3389/fneur.2019.01165 PMC6848258

[39] Storelli L et al (2022) Advanced diffusion-weighted imaging models better characterize white matter neurodegeneration and clinical outcomes in multiple sclerosis. J Neurol 269(9):4729–4741 35397753 10.1007/s00415-022-11104-z

[40] Koubiyr I et al (2024) Longitudinal fibre-specific white matter damage predicts cognitive decline in multiple sclerosis. Brain Commun 6(1):fcae018 38344654 10.1093/braincomms/fcae018 PMC10853982

[41] Zivadinov R et al (2018) Effect of dimethyl fumarate on gray and white matter pathology in subjects with relapsing multiple sclerosis: a longitudinal study. Eur J Neurol 25(3):584-e36 29316038 10.1111/ene.13562

[42] Zivadinov R et al (2018) Effect of teriflunomide on gray and white matter brain pathology in multiple sclerosis using volumetric and diffusion-tensor imaging MRI measures. J Neurol Sci 388:175–181 29627017 10.1016/j.jns.2018.03.028

