# Supplementary material for: Effects of cognitive rehabilitation and exercise on brain structure in progressive multiple sclerosis: results from the CogEx trial
Source: J Neurol. 2025 Sep 23;272(10):645. doi: 10.1007/s00415-025-13382-9 (PMC12457549; doi:10.1007/s00415-025-13382-9)
Supplement: Supplementary file 1 — Supplementary file1 (DOCX 28 KB) [file 415_2025_13382_MOESM1_ESM.docx]

**SUPPLEMENTARY TABLES**

**Supplementary Table 1.** Correlations between longitudinal volumetric differences and changes in cognitive tests at week 12 vs baseline. Results analyzed at cluster extent threshold=10 voxels, p<0.001 uncorrected and p<0.05 FWE-corrected.

| Measure | Group | kE | pFWE | r | MNIx | MNIy | MNIz | BA | Area |
| --- | --- | --- | --- | --- | --- | --- | --- | --- | --- |
| SDMT | CR+EX-sham | 24 | 0.962 | 0.42 | -62 | -21 | -28 | 20 | L ITG |
| zBVMT | CR+EX-sham | 172 | 0.613 | -0.49 | 48 | -66 | 0 | 37 | R MTG |
|  |  | 20 | 0.974 | -0.44 | 26 | -76 | 33 | 19 | R SOG |
|  |  | 22 | 0.993 | -0.43 | 40 | -72 | -36 | - | R Cerebellum Crus I |
| zCVLT | CR+EX-sham | 37 | 0.97 | 0.42 | 12 | -96 | -12 | 18 | R Lingual Gyrus |
|  |  | 30 | 0.978 | 0.41 | 36 | -70 | -14 | 19 | R Fusiform Gyrus |
|  |  | 19 | 0.983 | 0.41 | 26 | -14 | -21 | - | R Hippocampus |
|  |  | 70 | 0.449 | -0.48 | -45 | -20 | -32 | - | L ITG |
|  |  | 56 | 0.475 | -0.47 | -48 | 4 | -18 | 21 | L MTG |
|  |  | 17 | 0.819 | -0.44 | -10 | 40 | -27 | 11 | L Superior OFC |
|  | CR+EX | 36 | 0.947 | -0.51 | 10 | -58 | -45 | - | R Cerebellum Lobule VIII |
|  |  | 33 | 0.961 | -0.50 | -20 | -33 | -20 | - | L Cerebellum Lobule IV/V |
|  |  | 164 | 0.993 | -0.48 | 24 | -64 | -33 | - | R Cerebellum Crus I |
|  |  | 10 | 0.998 | -0.48 | -52 | -57 | -21 | 37 | L ITG |

Abbreviations: BA, Brodmann Area; BVMT, Brief Visuospatial Memory Test; CR, Cognitive rehabilitation; CR-sham, Sham cognitive rehabilitation; CVLT, California Verbal Learning Test; EX, Aerobic exercise; EX-sham, Sham exercise; ITG, Inferior Temporal Gyrus; kE, Cluster extent; L, Left; MTG, Middle Temporal Gyrus; OFC, Orbitofrontal Cortex; R, Right; SDMT, Symbol Digit Modalities Test; SOG, Superior Occipital Gyrus.

**Supplementary Table 2.** Correlations between longitudinal volumetric differences and changes in cognitive tests at month 9 vs week 12. Results analyzed at cluster extent threshold=10 voxels, p<0.001 uncorrected and p<0.05 FWE-corrected.

| **Measure** | **Group** | **kE** | **pFWE** | **r** | **MNIx** | **MNIy** | **MNIz** | **BA** | **Area** |
| --- | --- | --- | --- | --- | --- | --- | --- | --- | --- |
| SDMT | CR-sham+  EX-sham | 23 | 0.56 | 0.43 | 16 | 3 | -30 | - | R PHG |
|  |  | 12 | 0.935 | 0.38 | 36 | 20 | -30 | 38 | R STP |
|  |  | 18 | 0.977 | 0.37 | -30 | -75 | -32 | - | L Cerebellum Crus I |
|  | EX+CR-sham | 241 | 0.123 | 0.50 | 34 | -88 | 20 | 19 | R MOG |
|  |  | 80 | 0.541 | 0.45 | -26 | -87 | 26 | 19 | L SOG |
|  |  | 131 | 0.322 | -0.47 | -30 | 38 | -10 | 11 | L Middle OFC |
|  |  | 113 | 0.546 | -0.45 | -30 | -40 | -30 | - | L Cerebellum Lobule VI |
|  |  | 96 | 0.921 | -0.41 | -60 | -32 | -2 | 21 | L MTG |
|  |  | 115 | 0.947 | -0.40 | 4 | 50 | -6 | 10 | R Middle OFC |
|  | CR+EX | 1054 | 0.053 | 0.61 | -38 | -14 | -21 | 20 | L Fusiform Gyrus |
|  |  | 98 | 0.898 | 0.49 | -45 | -51 | -42 | - | L Cerebellum Crus II |
| zBVMT | CR-sham+  EX-sham | 22 | 0.901 | -0.40 | -30 | -98 | 6 | - | L MOG |
|  | EX+CR-sham | 16 | 0.906 | 0.42 | 3 | -54 | 42 | - | R Precuneus |
|  |  | 33 | 0.737 | -0.44 | 33 | 60 | -2 | 11 | R Middle OFC |
|  |  | 20 | 0.775 | -0.44 | -39 | -42 | -27 | - | L Cerebellum Lobule VI |
|  | CR+EX-sham | 207 | 0.717 | -0.50 | 20 | -38 | -20 | - | R Cerebellum Lobule IV/V |
|  | CR+EX | 47 | 0.878 | -0.49 | 62 | -3 | 22 | 43 | R Postcentral Gyrus |
| zCVLT | CR-sham+  EX-sham | 74 | 0.427 | 0.44 | 54 | 38 | 2 | 45 | R IFG Pars Triangularis |
|  |  | 71 | 0.804 | 0.41 | 14 | -80 | -2 | 18 | R Calcarine Sulcus |
|  | EX+CR-sham | 56 | 0.596 | 0.45 | 9 | -56 | 15 | 30 | R Calcarine Sulcus |
|  | CR+EX-sham | 14 | 0.996 | -0.45 | 30 | -45 | -34 | - | R Cerebellum Lobule VI |
|  | CR+EX | 28 | 0.913 | 0.49 | -46 | -28 | -24 | 20 | L ITG |
|  |  | 32 | 0.963 | -0.47 | 44 | -57 | -21 | 37 | R Fusiform Gyrus |

Abbreviations: BA, Brodmann Area; BVMT, Brief Visuospatial Memory Test; CR, Cognitive rehabilitation; CR-sham, Sham cognitive rehabilitation; CVLT, California Verbal Learning Test; EX, Aerobic exercise; EX-sham, Sham exercise; IFG, Inferior Frontal Gyrus; ITG, Inferior Temporal Gyrus; kE, Cluster extent; L, Left; MOG, Middle Occipital Gyrus; MTG, Middle Temporal Gyrus; OFC, Orbitofrontal Cortex; PHG, Parahippocampal Gyrus; R, Right; SDMT, Symbol Digit Modalities Test; SOG, Superior Occipital Gyrus; STP, Superior Temporal Pole.
