# Supplementary material for: Effects of cognitive rehabilitation and exercise on brain structure in progressive multiple sclerosis: results from the CogEx trial
Source: J Neurol. 2025 Sep 23;272(10):645. doi: 10.1007/s00415-025-13382-9 (PMC12457549; doi:10.1007/s00415-025-13382-9)
Supplement: Supplementary file 2 — Supplementary file1 (DOCX 21 KB) [file 415_2025_13382_MOESM2_ESM.docx]

**CogEx Research Team Coinvestigators**

| **Name** | **Location** | **Role** | **Contribution** |
| --- | --- | --- | --- |
| Anne Sophie Michelsen | Aarhus University | Research Assistant | Data acquisition, and data entry |
| Laurits Emil Taul Madsen | Aarhus University | Physiotherapist | Quality control, data acquisition, and data entry |
| Marie-Louise Kjeldgaard Jørgensen | Aarhus University | Lab Coordinator | Quality control |
| Mette Dahl Diedmann | Aarhus University | Research Assistant | Data acquisition |
| Charly Keytsman | Hasselt University | Research Assistant | Data acquisition |
| Ellen Vanzeir | Hasselt University | Research Assistant | Data acquisition, and data entry |
| Joke Lenaerts | Hasselt University | Research Assistant | Data acquisition |
| Leen Knevels | Hasselt University | Research Assistant | Data acquisition |
| Mieke D'Hooge | Hasselt University | Lab Coordinator | Data acquisition, data entry |
| Natasja De Weerdt | Hasselt University | Research Assistant | Data acquisition, and data entry |
| Renee Veldkamp | Hasselt University | Lab Coordinator | Data acquisition, and data entry |
| Rudi Donnee | Hasselt University | Person living with multiple sclerosis | CogEx team advisor |
| Séline Vandecasteele | Hasselt University | Research Assistant | Data acquisition |
| Veerle Vandael | Hasselt University | Research Assistant | Data acquisition, and date entry |
| Claudio Cordani | IRCCS San Raffaele Scientific Institute, Milano | Physiotherapist | Data acquisition, and data entry |
| Paola Valsasina | IRCCS San Raffaele Scientific Institute, Milano | Biotechnology Technician | MRI data analysis |
| Carmen Vizzino | IRCCS San Raffaele Scientific Institute, Milano, Italy | Neuropsychologist | Data acquisition, and data entry |
| Nicolò Tedone | IRCCS San Raffaele Scientific Institute, Milano, Italy | Neuropsychologist | Data acquisition, and data entry |
| Paolo Preziosa | IRCCS San Raffaele Scientific Institute, Milano, Italy | Neurologist | Patient recruitment |
| Jessica Podda | Italian MS Foundation | Research Assistant | Data acquisition, and data entry |
| Ludovico Pedullà | Italian MS foundation | Physiotherapist | Data entry, and acquisition |
| Andrea Tacchino | Italian Multiple Sclerosis Foundation | Lab Coordinator | Patient recruitment |
| Angela Smith | Kessler Foundation | Lab Coordinator | Patient recruitment, and data entry |
| Blake Bichler | Kessler Foundation | Research Assistant | Data entry |
| Jimmy Morecraft | Kessler Foundation | Research Coordinator | Data acquisition and data entry |
| Michael DiBenedetto | Kessler Foundation | Research Assistant | Data acquisition, and data entry |
| Nancy Moore | Kessler Foundation | Lab Manager | Quality control |
| Catherine Holme | Plymouth University | Occupational Therapist | Quality control, data acquisition |
| Chris Cole | Plymouth University | Person living with multiple sclerosis | CogEx team advisor |
| Kimberley Algie | Plymouth University | Research Assistant | Data acquisition |
| Sara Chatfield | Plymouth University | Physiotherapist | Quality analysis, prepared CogEx conference posters |
| Juliana Puopolo | Sunnybrook Hospital | Physiotherapist | Data acquisition, and data entry |
| Laura Kenton | Sunnybrook Hospital | Research Assistant | Data acquisition, and data entry |
| Laura Toll | Sunnybrook Hospital | Person living with multiple sclerosis | CogEx team advisor |
| Ashlie Kristin Ithurburn | The University of Alabama at Birmingham | Research Physiotherapist | Data entry |
| Brendon Truax | The University of Alabama at Birmingham | Research Assistant | Data acquisition |
| Catherine Danielle Jones | The University of Alabama at Birmingham | Physiotherapist | Data acquisition, and data entry |
| Jessica Baird | The University of Alabama at Birmingham | Physiotherapist | Data acquisition, and data entry |
| Petra Silic | The University of Alabama at Birmingham | Lab Coordinator | Data acquisition, and data entry |
| Michelle Koch | University College London Hospital | Research Assistant | Data acquisition, and data entry |
| Patrizia Pajak | University College London | Research Assistant | Data acquisition, and data entry |
| Alexsandra Pietrusz | University College London Hospital | Research Physiotherapist | Data acquisition, and data entry |
| Catherine Smith | University College London Hospital | Research Assistant | Data acquisition |
| Holly Wilkinson | University College London Hospital | Research Assistant | Data acquisition, and data entry |
| James Braisher | University College London Hospital | Research Assistant | Data acquisition |
| Marie Braisher | University College London Hospital | Lab Manager | Quality control |
| Rebecca Bex Walters | University College London Hospital | Research Assistant | Data acquisition, and data entry |
| Claudia Niccolai | University of Florence | Research Assistant | Data acquisition, and data entry |
| Guido Pasquini | University of Florence | Research Assistant | Data Acquisition, and data entry |
| Irene Mosca | University of Florence | Research Assistant | Data acquisition, and data entry |
| Sara Della Bella | University of Florence | Research Assistant | Data acquisition, and date entry |
| Fedrica Vannetti | University of Florence, Italy | Lab Supervisor | Patient recruitment |
| Filippo Gerli | University of Florence, Italy | Physiotherapist | Data entry, and data entry |
| Chiara Pollio | University of Genoa | Physiotherapist | Data acquisition, and data entry |
| Eleonora Colombo | University of Genoa | Psychologist | Data acquisition, and data entry |
| Elisa Pelosin | University of Genoa | Research Assistant | Data acquisition, and data entry |
| Maria Cellerino | University of Genoa | Neurologist | Quality control |
| Matteo Pardini | University of Genoa | Research Assistant | Data acquisition |
| Roberto Hernandez | UT Southwestern Medical Center | Assistant Statistician | Data analysis |
| Michele Curran | Washington University School of Medicine in St. Louis | Lab Manager | Quality control, and data analysis |
